# Urbanization and Altitude Are Associated with Low Kidney Function in Peru

**DOI:** 10.1089/ham.2018.0106

**Published:** 2019-06-21

**Authors:** Rodrigo M. Carrillo-Larco, J. Jaime Miranda, Robert H. Gilman, Offdan Narvaez-Guerra, Karela Herrera-Enriquez, Josefina Medina-Lezama, Liam Smeeth, William Checkley, Antonio Bernabe-Ortiz

**Affiliations:** ^1^ Department of Epidemiology and Biostatistics, School of Public Health, Imperial College London, London, United Kingdom.; ^2^ CRONICAS Center of Excellence in Chronic Diseases, Universidad Peruana Cayetano Heredia, Lima, Peru.; ^3^ Department of Medicine, School of Medicine, Universidad Peruana Cayetano Heredia, Lima, Peru.; ^4^ Department of International Health, Bloomberg School of Public Health, Johns Hopkins University, Baltimore, Maryland.; ^5^ Área de Investigación y Desarrollo, Asociación Benéfica PRISMA, Lima, Peru.; ^6^ Department of Preventive Medicine, Integral Occupational Medicine Center CEMOIN, Arequipa, Peru.; ^7^ Universidad Católica de Santa María, Arequipa, Peru.; ^8^ Faculty of Epidemiology and Population Health, London School of Hygiene and Tropical Medicine, London, United Kingdom.; ^9^ Division of Pulmonary and Critical Care, School of Medicine, Johns Hopkins University, Baltimore, Maryland.

**Keywords:** altitude, chronic kidney disease, glomerular filtration, kidney function, Peru, urbanization

## Abstract

***Background:*** Kidney health needs to be studied in low- and middle-income countries with populations living at high altitude and undergoing urbanization. We studied whether greater level of urbanization was associated with worse kidney function and higher hemoglobin was associated with worse kidney function at high altitude.

***Methods:*** Cross-sectional analysis of population-based studies in Peru including five sites at different altitude above the sea level and urbanization level (in decreasing order of urbanization): Lima (sea level), Arequipa (2335 m), urban Puno (3825 m), Tumbes (sea level), and rural Puno (3825 m). The exposures were urbanization and altitude as per study site, and hemoglobin (g/dL). The outcome was the estimated glomerular filtration rate (eGFR).

***Results:*** Four thousand two hundred eight people were studied: mean age was 57.4 years (standard deviation: 12.4) and 51.9% were women. In comparison to rural Puno, eGFR was similar in Lima; in comparison to rural Puno, Arequipa, urban Puno, and Tumbes had worse eGFR, for example, in Arequipa, β = −8.07 (95% confidence interval [CI]: −10.90 to −5.24). Intermediate (β = −8.60; 95% CI: −10.55 to −6.66) and high (β = −11.21; 95% CI: −14.19 to −8.24) altitude were negatively correlated with eGFR when only urban places were analyzed. At high altitude, there was a trend for a negative association between hemoglobin and eGFR: β = −1.09 (95% CI: −2.22 to 0.04).

***Conclusions:*** Apparently, higher altitude and level of urbanization, except for one highly urbanized site, were associated with worse kidney function. Our findings suggest that some of the adverse impact of high altitude on kidney function has been balanced by the lower risk conferred by rural environments.

## Introduction

Despite the high global burden of chronic kidney disease (GBD 2016 Risk Factors Collaborators, 2017a, 2017c), kidney health remains understudied in low- and middle-income countries (LMICs), many of which host populations living at high altitude (Cohen and Small, 1998; worldatlas). In addition to risk factors for impaired kidney health associated with urbanization being experienced by LMICs (e.g., diabetes or hypertension), high altitude also imposes physiological challenges (e.g., chronic hypoxia) that influence kidney function (Luks et al., 2008; Arestegui et al., 2011). Therefore, studying kidney function across altitude and urbanization levels could inform where kidney health needs interventions to prevent diseases.

Erythropoiesis is associated with both kidney function and high altitude. At high altitude, greater hemoglobin would be expected; conversely, in the presence of kidney damage, hemoglobin would be expected to fall. Because people with impaired kidney function could response better to erythropoietin needing smaller doses to maintain high hematocrit, it has been reported that, at high altitude, people with impaired kidney function would have higher hemoglobin than people with normal kidney function (Hurtado-Arestegui et al., 2018). Further verification of this finding with a large population-based sample at different altitudes above the sea level could strengthen this observation and thus inform future studies and interventions.

To study the association between urbanization as well as high altitude and kidney function, and the association between hemoglobin and kidney function at high altitude, we tested these hypotheses: (1) greater level of urbanization would be associated with worse kidney function and (2) higher hemoglobin would be associated with worse kidney function at high altitude.

## Materials and Methods

### Study design and setting

This is a cross-sectional study using data of the PREVENCION study (Medina-Lezama et al., 2005) and the second follow-up round of the CRONICAS Cohort Study (Miranda et al., 2012). These are population-based studies conducted in 2004–2006 and 2013–2014, respectively. The PREVENCION study was conducted in Arequipa at 2335 m above the sea level. The CRONICAS Cohort Study was conducted in Lima, Tumbes, rural Puno, and urban Puno; Lima and Tumbes are at sea level, whereas both Puno sites are at 3825 m above the sea level. These five study sites have different degree of urbanization: Lima is highly urbanized followed by Arequipa, urban Puno, Tumbes, and rural Puno (Medina-Lezama et al., 2005; Miranda et al., 2012). To classify the study sites according to the degree of urbanization, the urbanicity scale adapted from Dahly and Adair (2007) was used.

### Population

People in the PREVENCION study were sampled following a probabilistic multistage approach. Stratification was based on socioeconomic status (i.e., household sanitation and access to basic services) and geographic location (i.e., the city was divided into clusters of 50 blocks and these further divided into aggregates of roughly 150 households each). The sampling aimed to include more than 200 people in each age group (15-year age groups from 20 years old up to age 80) (Medina-Lezama et al., 2005). For this work, people in the PREVENCION study were excluded if they had missing creatinine test (*n* = 1). For comparison purposes with the CRONICAS Cohort Study, people aged <35 years were excluded (*n* = 423). From the initial sample size of 2106, we studied 1682 people.

Participants in the CRONICAS Cohort Study were randomly selected following a sex- and age-stratified technique (10-year age groups from age 35 and 65+ years). The sampling frame was based on a census of each study site. Pregnant women, people with pulmonary tuberculosis, and those with disabilities that could have prevented fulfilling the clinical evaluation were excluded (Miranda et al., 2012). The CRONICAS Cohort Study included 3601 people at baseline, though creatinine tests were only collected in the second follow-up visit. We excluded people who had missing creatinine values (*n* = 1067). Individuals with missing information to compute the glomerular filtration rate (eGFR) were also excluded (*n* = 5). This process left 2526 people for the analysis.

The total sample size included in the analysis was 4208 people: 905 in Lima, 1682 in Arequipa, 390 in urban Puno, 901 in Tumbes, and 330 in rural Puno.

### Variables

Data collection of the CRONICAS Cohort Study and the PREVENCION study has been detailed elsewhere (Medina-Lezama et al., 2005; Miranda et al., 2012). In brief, data collection was conducted with paper-based questionnaires applied by trained fieldworkers, who also conducted the clinical evaluation including weight, height, and blood pressure (Chobanian et al., 2003). Blood samples for hemoglobin, creatinine, and fasting glucose were taken by trained technicians after an overnight fasting period (Medina-Lezama et al., 2005; Miranda et al., 2012). All blood samples of the PREVENCION study were analyzed by one laboratory, and so were blood samples of the CRONICAS Cohort Study.

Hypertension was defined as measured raised blood pressure (≥140/90 mmHg), self-reported diagnosis and currently under treatment. Diabetes was defined as fasting plasma glucose ≥126 mg/dL or self-reported diagnosis. Body mass index (BMI) was calculated using measured weight and height as kg/m^2^. Excessive erythrocytosis was defined as hemoglobin ≥21 and ≥19 g/dL for men and women, respectively (Leon-Velarde et al., 2005). Education level had four categories: none, primary education, secondary education, and higher education.

The outcome was eGFR as per the Modification of Diet in Renal Disease (MDRD) equation [units: mL/(min ·1.73 m^2^)] (National Institute of Diabetes and Digestive and Kidney Diseases); for descriptive purposes, eGFR was divided into three categories: <60, 60–89, and ≥90. The three predictors of interest were as follows: (1) study site and thus the degree of urbanization (Lima > Arequipa > urban Puno > Tumbes > rural Puno); (2) altitude above the sea level (low altitude [Lima and Tumbes], intermediate altitude [Arequipa], and high altitude [both Puno sites]); and (3) hemoglobin level in g/dL, which was further dichotomized at 13 and 12 g/dL for men and women, respectively (Organización Mundial de la Salud).

### Statistical analysis

Analysis was conducted with STATA 13.0 (StataCorp, College Station, TX). Means, standard deviations (SDs), and relative frequencies were estimated for descriptive purposes. Comparisons between categorical variables were conducted with the chi-square test. Comparison between numerical variables across levels of a categorical variable was performed with the analysis of variance test; the Bonferroni test was used to assess pairwise differences.

Linear regression models including robust standard errors were fitted to address the associations of interest: (1) eGFR and urbanization; (2) eGFR and altitude above the sea level; (3) eGFR and altitude among urbanized sites only (i.e., excluding Tumbes and rural Puno) to verify if the effect of altitude is similar across alike places; and (4) eGFR and hemoglobin by altitude levels. All models were presented as crude and adjusted. The first three models included these covariates: sex, age (numeric), education, hypertension, diabetes, BMI (numeric), height (numeric), and hemoglobin; the last model included these covariates except hemoglobin, which was the exposure of interest.

### Ethics

All participants gave an informed consent, and both studies were approved by institutional review boards: Universidad Peruana Cayetano Heredia (Lima, Peru) and Johns Hopkins University (Baltimore, Maryland) approved the CRONICAS Cohort Study; the Santa Maria Catholic University Human Research Committee (Arequipa, Peru) approved the PREVENCION study (Medina-Lezama et al., 2005; Miranda et al., 2012).

## Results

### Study sample

The mean age was 57.4 years (SD: 12.4), ranging from 35 to 94 years; there were more women (51.9%). The study population was homogenous regarding socio-demographic characteristics except for education: rural Puno had more people with no formal education (7.6%, *p* < 0.001). There were fewer anemia cases at high altitude: 1.0% and 0.6% in urban and rural Puno (*p* < 0.001), respectively; these figures for excessive erythrocytosis were 5.6% and 2.1% (*p* < 0.001, Supplementary Table S1).

### eGFR variation according to study site and altitude

**FIG. 1.**
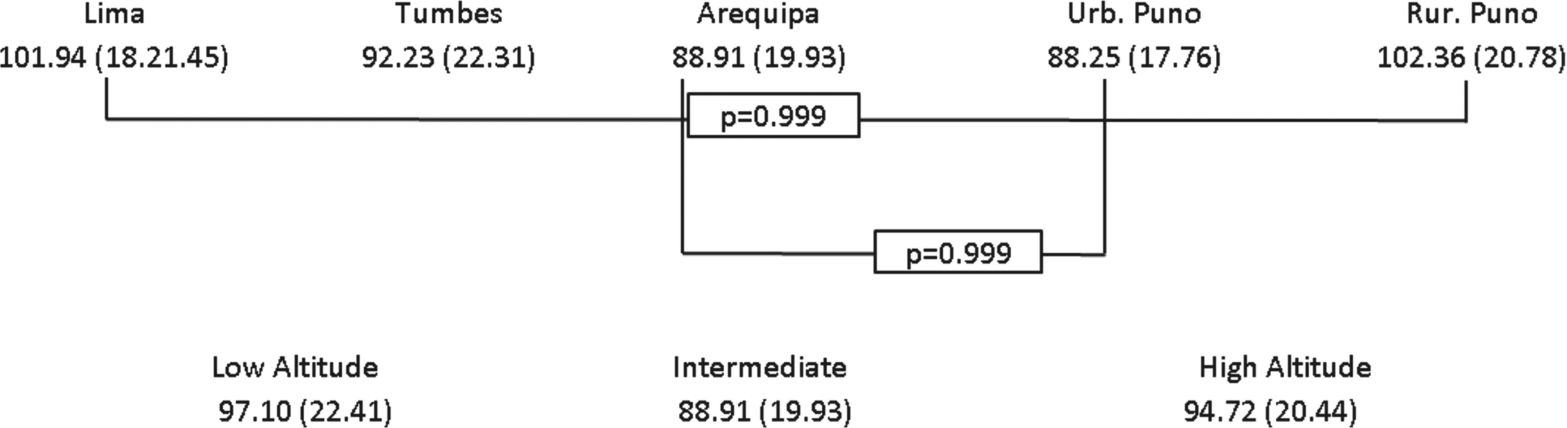
Mean (SD) eGFR as per the MDRD equation according to site and altitude. *p*-Values correspond to the Bonferroni multiple comparison test (analysis of variance). Pairwise relationships where no *p*-values are shown were *p* < 0.050. eGFR, estimated glomerular filtration rate; MDRD, Modification of Diet in Renal Disease; SD, standard deviation.

**FIG. 2.**
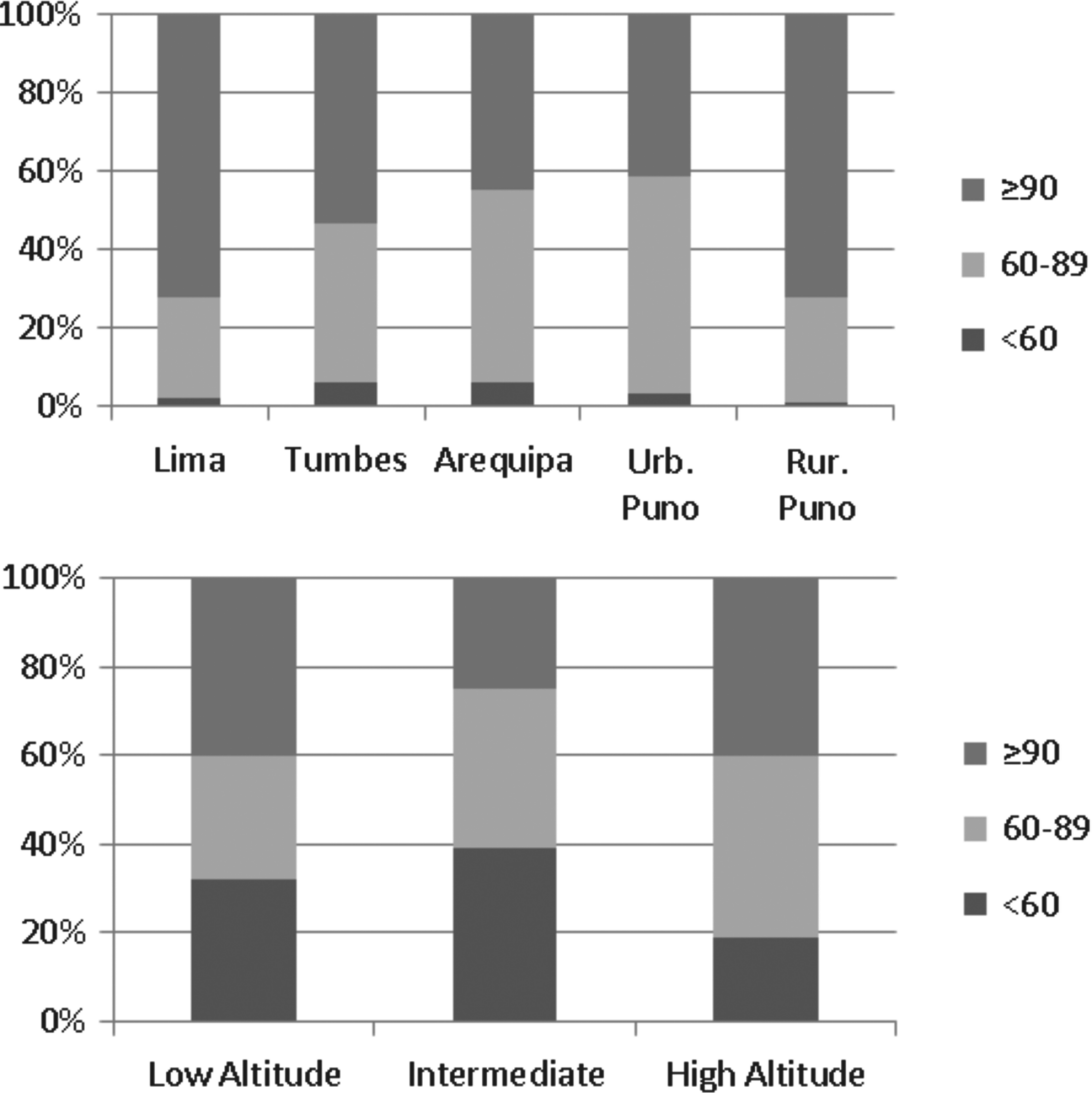
Proportion of people in each eGFR category according to study site and altitude.

Figure 1 shows the mean eGFR according to study site and altitude; Supplementary Table S2 shows eGFR box plots by study site. Lima and rural Puno had similar eGFR (≈102); accordingly, these study sites also had similar creatinine values (Supplementary Table S2). More than 20% of the study population across sites had an eGFR between 60 and 89, and the largest proportion of individuals with an eGFR ≥90 was found in Lima and rural Puno (Fig. 2). There seemed to be a negative association between eGFR and urbanization (Table 1): for example, in comparison to rural Puno, people in Arequipa would have in average −8.07 (95% confidence interval [CI]: −10.90 to −5.24) eGFR units. According to the results presented in Figure 1, there was not a strong difference between rural Puno and Lima (Table 1).

**Table 1.** Association Between Estimated Glomerular Filtration Rate [mL/(min·1.73 m^2^)] and Study Site As Well As Altitude Above the Sea Level

	*Crude*	*Model A*	*Model B*
	*β (95% CI)*	*β (95% CI)*	*β (95% CI)*
Study site (degree of urbanization)	*N* = 4208	*N* = 4101	*N* = 4046
Rural Puno	1	1	1
Tumbes (†)	**−10.13 (−12.80 to −7.46)**	**−7.12 (−9.92 to −4.32)**	**−7.16 (−10.33 to −3.99)**
Urban Puno (††)	**−14.11 (−16.96 to −11.26)**	**−10.83 (−13.91 to −7.74)**	**−10.78 (−13.88 to −7.69)**
Arequipa (†††)	**−13.45 (−15.89 to −11.02)**	**−8.22 (−10.92 to −5.53)**	**−8.07 (−10.90 to −5.24)**
Lima (††††)	−0.42 (−3.06 to 2.22)	0.31 (−2.48 to 3.09)	0.31 (−2.89 to 3.51)
Altitude above the sea level	*N* = 4208	*N* = 4101	*N* = 4046
Low altitude	1	1	1
Intermediate altitude	**−8.19 (−9.59 to −6.78)**	**−4.26 (−5.70 to −2.81)**	**−3.93 (−5.55 to −2.32)**
High altitude	**−2.38 (−4.19 to −0.56)**	**−2.53 (−3.34 to −0.71)**	**−**2.13 (**−**4.63 to 0.37)
Altitude above the sea level among urban sites	*N* = 2977	*N* = 2894	*N* = 2839
Low altitude	1	1	1
Intermediate altitude	**−13.03 (−14.72 to −11.34)**	**−8.66 (−10.43 to −6.88)**	**−8.60 (−10.55 to −6.66)**
High altitude	**−13.69 (−15.94 to −11.44)**	**−11.08 (−13.33 to −8.82)**	**−11.21 (−14.19 to −8.24)**

Model A includes sex, age (numeric variable), education, hypertension, diabetes, BMI (numeric variable), and height (numeric variable), whereas model B also includes hemoglobin (numeric variable). Estimates in bold are statistically significant (*p* < 0.050). Interpretation: in comparison to being a resident of rural Puno or of a low altitude site, the eGFR is expected to be higher or lower in a magnitude equal to the β value. Higher degree of urbanization is represented by the number of crosses (†). The regression model for altitude above the sea level among urban sites excluded Tumbes and rural Puno.

95% CI, 95% confidence interval; BMI, body mass index; eGFR, estimated glomerular filtration rate.

Regarding altitude above the sea level, eGFR did not seem to follow a positive linear relationship with the smallest eGFR found at intermediate altitude (Fig. 1). A similar proportion of subjects with an eGFR ≥90 was found at low and high altitude (Fig. 2). The adjusted regression model depicted lower eGFR at intermediate altitude (β = −3.93; 95% CI: −5.55 to −2.32) versus low altitude when all sites were included; when this model was restricted to urban places alone, that is, Tumbes and rural Puno were excluded, there was lower eGFR at intermediate (β = −8.60; 95% CI: −10.55 to −6.66) and high altitude (β = −11.21; 95% CI: −14.19 to −8.24, Table 1).

### The association between hemoglobin and eGFR

**FIG. 3.**
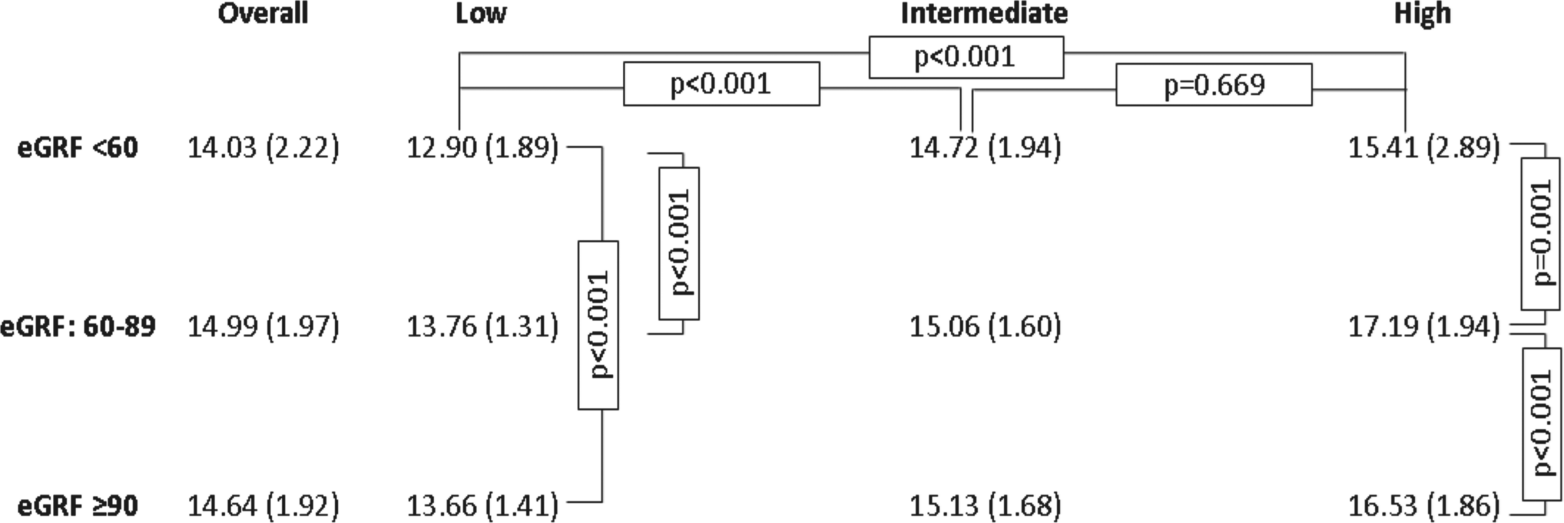
Mean (SD) hemoglobin according to eGFR categories and altitude above the sea level. *p*-Values correspond to the Bonferroni multiple comparison test (analysis of variance). By *column*, pairwise relationships where no *p*-values are shown were *p* > 0.050. By *row*, all pairwise relationships were different at *p* < 0.001 (only *first row* showed for simplicity).

Figure 3 signals that, regardless of the eGFR category, people living at high altitude had higher hemoglobin than people at sea level. Within each level of altitude, most comparisons of mean hemoglobin across eGFR categories were nonsignificant, suggesting that people with an eGFR <60 had similar hemoglobin to a person with an eGFR ≥90 within each level of altitude.

The adjusted regression models suggest that there was a negative weak association between hemoglobin and eGFR levels at high altitude: for each additional hemoglobin unit, the eGFR could have been expected to fall by −1.09 (95% CI: −2.22 to 0.04) units (Table 2). To understand whether this negative association existed regardless of the overall hemoglobin level, we stratified the regression model at high altitude by hemoglobin tertile (first tertile bottom). These stratified models did not yield any strong associations in the first and second tertile, although in the top tertile, the association was stronger than the one already reported: −2.52 (95% CI: −3.63 to −1.41), suggesting that at high altitude and among individuals with high hemoglobin, the hemoglobin level is negatively associated with the eGFR.

**Table 2.** Association Between Hemoglobin (g/dL) and Estimated Glomerular Filtration Rate [mL/(min·1.73 m^2^] According to Altitude Above the Sea Level

	*Overall*	*Low*	*Intermediate*	*High*
	*β (95% CI)*	*β (95% CI)*	*β (95% CI)*	*β (95% CI)*
	Crude			
	*N* = 4125	*N* = 1806	*N* = 1599	*N* = 720
Hemoglobin	**−0.55 (−0.91 to −0.20)**	−0.40 (−1.34 to 0.54)	**0.75 (0.16 to 1.34)**	**−1.20 (−2.02 to −0.38)**
	Adjusted			
	*N* = 4046	*N* = 1794	*N* = 1563	*N* = 689
Hemoglobin	−0.12 (−0.62 to 0.39)	−0.19 (−1.29 to 0.91)	0.30 (−0.33 to 0.94)	−1.09 (−2.22 to 0.04)

Adjusted models include sex, age (numeric variable), education, hypertension, diabetes, BMI (numeric variable), and height (numeric variable); the adjusted model for overall also include altitude above the sea level. Estimates in bold are statistically significant (*p* < 0.050). Interpretation: within each altitude category, for one additional hemoglobin unit, the eGFR changes (increases or decreases) in a magnitude equal to the β value.

## Discussion

### Main findings

The take-home message of this work is that in five Peruvian sites at different level of urbanization and altitude above the sea level there may be a negative association between greater urbanization and kidney function as per eGFR; nevertheless, these estimates need to be understood in context with the limitations of the study and with the fact that there was not a strong difference between the most and least urbanized study sites. However, the results also suggest that higher hemoglobin level is associated with worse eGFR in high altitude sites and among people with high hemoglobin; this finding needs to be interpreted accounting for the limitations of the study such as lack of adjustment for some potential confounders (e.g., diet). Overall, these results draw attention to kidney health in places undergoing urbanization, where most resources are being allocated to address other—important—noncommunicable diseases. These findings call to better understand the role of hemoglobin in kidney function at high altitude.

### Results interpretation: eGFR and urbanization

The first hypothesis was that greater level of urbanization would be associated with worse kidney function. Accounting for the limitations of the study, our results partially supported this hypothesis; although three urbanized places had lower eGFR than the least urban study site, the study site with the greatest level of urbanization had a similar eGFR as the least urbanized setting.

Our findings partially suggest that higher urbanization is associated with lower eGFR. This could be because of the high frequency of unhealthy lifestyles (e.g., sedentarism) and diseases (e.g., diabetes) in more urbanized places. Our results support this assumption: hypertension, diabetes, and obesity prevalence were the lowest in rural Puno. Obesity, diabetes, hypertension, and high cholesterol are on the rise across the world with larger burden in LMICs [NCD Risk Factor Collaboration (NCD-RisC), 2016, 2017a, 2017b]. Reducing these risk factors should be a priority not only because of their impact on cardiovascular health but also because of their relation with kidney health (Global Burden of Metabolic Risk Factors for Chronic Diseases Collaboration, 2014; GBD 2016 Risk Factors Collaborators, 2017b).

An unexpected finding of this work was the similar eGFR between Lima (sea level and highly urbanized) and rural Puno (high altitude and highly rural). The evidence suggesting that risk factors for impaired kidney function, namely diabetes and hypertension, are more prevalent in Lima versus rural Puno does not support this observation. In addition, contrary to what had been expected, our results also suggested that there could be higher eGFR in Tumbes (sea level) than urban Puno (high altitude), even though the prevalence of clinical risk factors for impaired eGFR was higher in Tumbes. Although largely speculative, we could argue that living at low altitude could delay, not necessarily prevent, renal damage in the presence of other relevant clinical risk factors for low eGFR (e.g., hypertension). Probably, the protective effect of low altitude could even outweigh the negative effects of established clinical risk factors. Longitudinal studies are needed to assess this hypothesis.

There could be other potential explanation for the similar eGFR between Lima and rural Puno. Lima was the study site with the second highest anemia prevalence. Anemia would trigger erythropoietin production (Jelkmann, 2011). Because erythropoietin may have renoprotective properties (Kuriyama et al., 1997; Tsubakihara et al., 2012; Covic et al., 2014), its enhanced production would prevent eGFR from decreasing. Nevertheless, although Tumbes was the site with the highest anemia prevalence and thus should have also had higher eGFR values, we argue that because Tumbes is also the site with the highest prevalence of other relevant clinical risk factors (e.g., diabetes and hypertension), the potential protective effect of erythropoietin was outweighed by the negative effects of the other risk factors. This last speculation would also explain the eGFR difference between rural and urban Puno. Although both are at high altitude hence in a hypoxemic environment which would increase erythropoietin production (Jelkmann, 2011), the fact that urban Puno has higher prevalence of diabetes and hypertension would explain the lower eGFR in comparison to rural Puno.

Finally, we could also argue that the lack of difference between Lima and rural Puno was due to confounding. In fact, we could argue that both Puno sites could have higher eGFR values. Puno sites had high prevalence of polycythemia hence the postcapillary oncotic pressure would be expected to be high. When the postcapillary oncotic pressure becomes similar to the hydrostatic pressure, then a decline in the glomerular filtration rate would be expected. Consequently, the eGFR in Puno sites could have been higher, so that rural Puno would have higher eGFR than Lima, perhaps even showing a strong difference.

### Results interpretation: eGFR and altitude

Our results suggest that there is a worse eGFR at high altitude above the sea level in comparison to low altitude. When all study sites were included, the effect of altitude on eGFR was larger at intermediate altitude than at high altitude; however, when only urban sites were included, there was a worse eGFR at intermediate and high altitude compared with low altitude. This suggests that the initial weak estimates at high altitude could have been confused by other features of urbanization, that is, different epidemiological profiles.

A small study (*n* = 293) with healthy individuals in La Paz, Bolivia (3640–4500 m above the sea level) and Lima, Peru, also concluded that people at high altitude have worse kidney function (Hurtado-Arestegui et al., 2018); of note, both were urbanized cities. Our results expand the available evidence because we included general population, thus accounting for generalizability of these findings; additionally, we included intermediate altitude suggesting that the negative association between altitude and eGFR also exists at around 2000 m above the sea level.

### Results interpretation: hemoglobin and eGFR at high altitude

The second hypothesis was that higher hemoglobin levels would be associated with lower eGFR at high altitude only. Our results partially supported this hypothesis; although the regression estimates were in the expected direction, the adjusted models did not show associations. Nonetheless, in a subgroup analysis including people with high levels of hemoglobin, the results did reveal a strong negative association between hemoglobin and eGFR.

Our results suggest that there is a negative association between hemoglobin and eGFR at high altitude, particularly among people with high hemoglobin. Similar findings were reported by Hurtado-Arestegui et al. (2018) in La Paz and Lima. Nevertheless, our results suggest that this negative association seems to be largely of high-altitude places and not so at intermediate altitude.

Hurtado-Arestegui et al. (2018) argued that worse kidney function at high altitude could be due to higher hemoglobin leading to less renal plasma flow (RPF), which in turns increases the kidney filtration fraction trying to keep the filtration rate constant (Lozano and Monge, 1965); this would eventually cause chronic kidney impairment (Brenner, 1983). Our results add to those reached by Hurtado-Arestegui et al. (2018) quantifying the association between hemoglobin and eGFR across different altitudes levels.

In addition to these mechanisms, the effect of chronic exposure to hypoxia should not be neglected. A misbalance in the action of hypoxia-inducible factors may impair kidney function—tubulointerstitial hypoxia—and lead to end-stage renal disease (Shoji et al., 2014). Therefore, this mechanism could also explain our findings.

### Implications for clinical practice

Our findings suggest that there could be a negative association between eGFR and hemoglobin at high altitude, which is likely to be because of a reduced RPF. Therefore, and according to each clinical scenario, one could try to avoid other actions that may further compromise the RPF, namely actions that would lead to a reduced blood flow (e.g., dehydration) or constraint of the afferent arterioles (e.g., use of nonsteroidal anti-inflammatory drugs). Conversely, it has been shown that angiotensin-converting enzyme inhibitors (ACEIs) may reduce hemoglobin level (Leshem-Rubinow et al., 2012); specifically at high altitude, ACEIs could reduce polycythemia and proteinuria (Plata et al., 2002). Our results provided evidence of places where these treatments could be further tested to benefit large populations.

In our study settings, an external factor that may also play a relevant role is cold (Broman et al., 1998; Yoon et al., 2014). Cold weather could increase blood viscosity and also vasoconstriction. In the last years, extreme cold temperatures have been experienced in Puno (reliefweb 2015; Vice News). Current evidence could suggest strengthening prevention strategies for kidney diseases at high altitude under extreme cold weather. Our results would suggest strengthening prevention strategies in high-altitude urbanized places.

Finally, it is relevant to pinpoint that these recommendations should be applied cautiously and specific to the population of interest. In other words, these recommendations may not apply to all individuals living at high altitude because they may not express the same health consequences. For example, people living in the Tibet, although exposed to high altitude, have normal levels of hemoglobin; this is believed to be due to adaptation over larger periods than the Andes habitants (Hurtado et al., 2012).

### Strengths and limitations

We studied a large sample size living at low, intermediate, and high altitude above the sea level, in places at different stage of urbanization. Notwithstanding, limitations should also be acknowledged. In addition to the limitations of the original data sources (Medina-Lezama et al., 2005; Miranda et al., 2012), this work has the following pitfalls. First, we relied on the MDRD equation to assess kidney function. The accuracy of this equation has not been tested in our population. However, this would be a nondifferential misclassification of the outcome variable, leading to a larger or smaller estimate effect of the regression models. Because the descriptive results show a consistent pattern with the regression models, the results provide evidence to support the conclusions. Second, we did not collect specific details about the laboratory procedures to analyze blood samples. Therefore, we cannot definitely rule out that some of the findings were due to technical issues, that is, differential misclassification. However, all samples of the CRONICAS Cohort Study were analyzed by one laboratory, and so were the samples of the PREVENCION study. If there were technical issues, these would have been reflected in the results between these studies but not when comparing sites within one study (e.g., between Lima and rural Puno, both of the CRONICAS Cohort Study). Third, due to data availability, kidney function was based on eGFR alone; therefore, our findings could suggest that there is even a larger population with some level of underlying kidney damage. In fact, other study in Peru revealed that proteinuria was the largest contributor to chronic kidney disease prevalence (Francis et al., 2015). In addition, the high altitude renal syndrome, which presents preserved kidney function (Arestegui et al., 2011), may have also hidden a larger prevalence of impaired kidney function. Fourth, variables addressing diet profiles were not available, so meat consumption, a relevant actor in creatinine metabolism, could not be adjusted for in the results; access to health care could have been a potential confounder too, although it was not available for adjustment. Fifth, BMI does not necessary represent muscle mass, which is also relevant in creatinine metabolism. We did not have a comprehensive measurement of muscle mass. However, in *post hoc* analysis and only for the CRONICAS Cohort Study because of data availability, we tested the fully adjusted regression models without BMI but including lean mass (kg). The results were virtually the same to the findings already shown; the only relevant difference was in the association between hemoglobin and eGFR at high altitude, which now depicted a strong negative association (−1.23; 95% CI: −2.24 to −0.22). This *post hoc* finding suggests that there is probably a negative association, but our main results were underpowered. Sixth, there was not an indicator of time exposed to each altitude level. This information would be relevant to further dissect the negative association between hemoglobin and eGFR at high altitude and to understand the effect of acute and chronic exposure to high altitude. Nevertheless, more than 65% of the population in urban Puno, Tumbes, and rural Puno has always lived in their study site, thus being chronically exposed to the corresponding altitude above the sea level. When the mean eGFR across study sites was estimated for those who reported to have always lived in the same place, the results presented in Figure 1 did not change substantially.

## Conclusions

It seems that higher altitude and higher level of urbanization, except for one highly urbanized site, were associated with worse kidney function. Our findings suggest that to date, some of the adverse impact of high altitude on kidney function has been balanced by the lower risk conferred by rural environments. However, increased urbanization at high altitude settings is likely to markedly increase the risk of chronic kidney disease among sizeable populations living at high altitude worldwide.

## Supplementary Material

Supplemental data

Supplemental data

## Data Availability

Data used to support the findings of this study are available from the corresponding author upon request.
