# Supplementary material for: Urbanization and Altitude Are Associated with Low Kidney Function in Peru
Source: High Alt Med Biol. 2019 Jun 21;20(2):133–40. doi: 10.1089/ham.2018.0106 (PMC6602110; doi:10.1089/ham.2018.0106)
Supplement: Supplemental data [file Supp_TableS1.pdf]

SUPPLEMENTARY MATERIAL

Supplementary Table 1: Characteristics of the study population according to study site

Variable	Lima	Arequipa	Urb. Puno	Tumbes	Rur. Puno	p-value
Degree or urbanization	+++++	++++	+++	++	+	
Sex	N=905	N=1,682	N=390	N=901	N=330	0.373
Women	52.6	53.4	50.0	49.8	50.0	
Men	47.4	46.6	50.0	50.2	50.0	
Age	N=905	N=1,682	N=390	N=901	N=330	0.444
Mean (SD)	57.1 (11.6)	57.3 (12.5)	57.0 (11.9)	58.1 (13.1)	57.3 (12.7)	
Has lived in this place, all your life?	N=1,095		N=762	N=1,030	N=699	<0.001
No	81.6		31.6	35.0	3.6	
Yes	18.4		68.4	65.0	96.4	
Education	N=904	N=1,658	N=390	N=900	N=330	<0.001
None	7.1	1.3	3.6	4.3	7.6	
Primary	33.4	14.5	8.2	49.6	49.4	
Secondary	41.8	36.3	25.6	31.2	34.9	
Higher	17.7	47.9	62.6	14.9	8.2	
Diabetes	N=903	N=1,653	N=389	N=901	N=330	<0.001
No	90.4	92.9	90.8	86.1	95.2	
Yes	9.6	7.1	9.2	13.9	4.9	
Hypertension	N=904	N=1,674	N=379	N=901	N=311	<0.001
No	78.9	74.3	85.8	63.0	90.0	
Yes	21.1	25.8	14.3	37.0	10.0	
SBP	N=904	N=1,672	N=379	N=901	N=311	<0.001
Mean (SD)	117.4 (18.1)	124.4 (20.6)	110.0 (17.3)	126.2 (21.3)	111.5 (16.2)	
DBP	N=904	N=1,672	N=379	N=901	N=311	<0.001

Mean (SD)	70.0 (10.4)	79.6 (9.3)	70.7 (10.8)	76.1 (10.9)	73.3 (9.8)	
BMI categories	N=901	N=1,673	N=379	N=897	N=311	<0.001
<25 Kg/m^2	22.2	29.3	21.6	21.0	45.7	
25-29.9 Kg/m^2	44.3	46.0	51.7	46.3	39.6	
≥30 Kg/m^2	33.5	24.7	26.7	32.8	14.8	
BMI	N=901	N=1,673	N=379	N=897	N=311	<0.001
Mean (SD)	28.5 (4.7)	27.4 (4.6)	28.1 (4.1)	28.5 (4.8)	25.9 (4.0)	
Anemia	N=905	N=1,599	N=390	N=901	N=330	<0.001
No	89.2	97.2	99.0	88.1	99.4	
Yes	10.8	2.8	1.0	11.9	0.6	
Excessive erythrocytosis	N=905	N=1,599	N=390	N=901	N=330	<0.001
No	100.0	99.1	94.4	100.0	97.9	
Yes	0.0	0.9	5.6	0.0	2.1	
Hemoglobin	N=905	N=1,599	N=390	N=901	N=330	<0.001
Mean (SD)	13.6 (1.4)	15.1 (1.7)	17.1 (2.1)	13.7 (1.4)	16.4 (1.8)	
Creatinine	N=905	N=1,682	N=390	N=901	N=330	<0.001
Mean (SD)	0.7 (0.2)	0.8 (0.2)	0.8 (0.3)	0.8 (0.3)	0.7 (0.1)	
eGFR categories	N=905	N=1,682	N=390	N=901	N=330	<0.001
<60	2.0	5.7	3.3	6.1	0.6	
60-89	25.8	49.5	55.4	40.3	27.3	
≥90	72.2	44.9	41.3	53.6	72.1	
